# Population Equation: Balancing What We Need With What We Have

**DOI:** 10.1289/ehp.113-a598

**Published:** 2005-09

**Authors:** Richard Dahl

Planet Earth, now home to about 6.5 billion human beings, has thus far disproved the doomsayers. In 1798, Rev. Thomas Robert Malthus predicted that population would outrun food supply on the assumption that human numbers would increase at a geometric rate while food would be limited to arithmetic increases. Then, in 1968, Stanford University professor Paul R. Ehrlich issued a similar warning in his book *The Population Bomb*, in which he predicted that hundreds of millions of people would die of starvation in the 1970s and 1980s.

Both men underestimated humanity’s resourcefulness—as well as its scientific and technological acumen—in figuring out how to provide for its growing numbers. Still, there’s little doubt that the Earth’s human carrying capacity has a limit. And growth can’t continue indefinitely without more of the significant environmental health impacts we are already seeing. In addition to documenting exactly how much growth is occurring, scientists are now interested in trends reflecting where such growth is occurring and the effect of factors such as consumption rates and migration on sustainability of the Earth’s resources.

## Maximum Capacity

Nobody really knows what the planet’s human carrying capacity is. Some, like Cornell University ecology and agriculture professor David Pimentel, contend that the Earth has already passed that point. Citing high malnutrition rates in the world, Pimentel estimates that the Earth’s carrying capacity—providing a quality life for all inhabitants—would appear to be about 2 billion. Other estimates go to both extremes. In a 1995 Cato Institute essay titled “The State of Humanity: Steadily Improving,” Julian L. Simon, the late University of Maryland economist, wrote, “We have in our hands now—actually in our libraries—the technology to feed, clothe and supply energy to an ever-growing population. . . . Even if no new knowledge were ever gained . . . we would be able to go on increasing our population forever.” On the other end of the spectrum, in 1971—three years after writing *The Population Bomb*—Ehrlich placed the limit at 500 million.

Others suggest that humans are already finding a way to take care of the population problem as evidenced by declining birth rates everywhere in the world. Declining birth rates don’t necessarily translate into declining populations, however. The United Nations (UN) Population Division projects that by 2050, global population could reach 9.1 billion.

This greater global population will differ from the current one in several ways. The population growth of the developed world has slowed to a crawl; fertility rates are on the decline and in some countries, such as Italy and Japan, population itself is projected to peak in five years. But poor countries will experience large increases for decades to come. Meanwhile, the UN points out that in 2007, for the first time in history, the global population will cross over from being predominantly rural to mostly urban, and that that trend will continue indefinitely.

“Most of the growth that’s going to happen in the next twenty, thirty years is going to be happening in the poor countries—it’s going to happen mostly in the cities, and mostly in the slums of the cities,” says John Bongaarts, vice president of policy research at the nonprofit Population Council. “Most of the next two or three billion people will end up in the slums of the poorest countries.”

Like many demographers, Bongaarts sees the decline in fertility rates, mostly in the industrial world, as the emerging worldwide norm. This means, he says, that at some point the poorer countries will reach the same stabilization point that the developed world has achieved and that global population will one day decline. He projects that peak will be reached at about 9.5 billion people.

Perhaps surprisingly, population’s relationship to health and environmental impacts is often ignored or glossed over by policy makers. In part, says Robert Engelman, vice president for research at the policy action group Population Action International, there’s a belief that “population will take care of itself.” But there’s also a reticence to talk about population because it gets tied up in politics, including the abortion debate.

Julie Starr, a population and environment specialist with the National Wildlife Federation, says she was surprised to see that the eight UN Millennium Development Goals that were set in 2000 failed to make any mention of population growth and family planning. These goals summarize all the development goals agreed to at international conferences and summits during the 1990s, with a target achievement date of 2015. “Each of the goals has specific targets, and population is mentioned nowhere—not even in goals that deal with maternal health and poverty,” she says. “Our message is that you can’t do anything about environmental sustainability if we don’t address population.”

“There’s been a lack of attention to the fact that population continues to grow in the world at a rate that is certainly unsustainable,” Engelman says. “And population is connected to environmental conditions everywhere. There really isn’t any environmental area that you can look at and say that it’s completely irrelevant to the number of people living in a particular ecosystem or watershed.”

## Marking the Trends

An international group of scientists who took part in a major new international study, however, apparently wants to see greater attention paid to population in future discussions about environmental sustainability. The Millennium Ecosystem Assessment, launched by UN secretary-general Kofi Annan in 2000 to assess the impact that environmental changes would have on achieving the Millennium Development Goals, involved the work of 1,360 scientific experts who aspired to measure the environmental impact that people are having on the Earth.

One document to emerge from the assessment process is *Ecosystems and Human Well-Being: Synthesis*, released in March 2005, which is one of several periodic reports scheduled for release through the end of 2005. This report examined the “services” that ecosystems provide (for example, fish from the ocean and pollution filtration from wetlands) and concluded that 15 of the 24 services are being degraded or used unsustainably. It suggested that the various environmental declines comprise a roadblock to achieving many of the Millennium Development Goals, including those calling for ensurance of global environmental stability, poverty alleviation, and food security.

The role of population in causing these declines is implicit throughout *Ecosystems and Human Well-Being: Synthesis* and explicit in a section in which it is identified as one of five “indirect drivers” that are altering ecosystems. Walter V. Reid, director of the assessment project, says ecosystem health is affected by two kinds of pressures that humans exert: changes in demand for (and consumption of ) an ecosystem’s specific services, and changes in emissions that might harm the ecosystem. “Obviously, both change in demand and change in emissions are closely tied to the combination of population change and economic growth,” he says.

To Reid, the most troubling development regarding population trends and their environmental impact is the fact that the greatest population growth is now occurring in environmentally fragile areas, like drylands and mountainous regions, where water is scarce and the soil is generally poor. In those areas, he says, “if you have high population growth that is overtaxing the capacity of the soils to provide food, you have high rates of soil erosion and depletion, and there’s just no buffer. And if you need more water, there’s just no buffer of water even to begin with.”

Demographers and social scientists use the term “poverty trap” to describe such areas, which are characterized by classic vicious cycles. “The pressure to degrade resources is insurmountable,” Reid says. “People don’t have other options. And when they degrade resources, that leads in the long run to higher levels of poverty and infant mortality and lower income, which leads to greater pressure to degrade resources.”

Another population trend emphasized in the March report is the movement of people to coastal areas around the world. Coastal ecosystems—marshes, mangroves, reefs—are extremely important contributors to human well-being, serving as breeding and nursery grounds for many species and as erosion prevention buffers between land and sea. Yet these benefactors are rapidly being destroyed. According to Reid, 35% of the world’s mangroves and 20% of its coral reefs have disappeared in the last two to three decades due to human pressure.

The assessment makes a variety of recommendations for policy makers—remove environmentally harmful subsidies to agriculture and fisheries, improve management of ecosystem services in regional planning decisions, provide public education about the importance of ecosystems, promote greener technologies, and more. But Reid believes that if the report is to have an impact, there must be some kind of repeating assessment process. He thinks that a mechanism should be created so that the subject is revisited in similar fashion every 10 years.

## The Role of Consumption

Roger-Mark De Souza, technical director of the Population, Health, and Environment program at the Population Reference Bureau, points out that another important trend in the developing world is its high and growing proportion of young people. In sub-Saharan Africa, for example, the proportion of people under 15 to people over 65 is 44% to 3%, according to the bureau. In Latin America, the numbers are 32% younger people compared to 6% older people. “That means that we will have continued population growth for some period of time because those young people of today are tomorrow’s parents,” he says. “We call that ‘population momentum.’”

In addition to their raw numbers, De Souza says, ever-increasing globalization means the growing ranks of young people in the developing world may be driven to consume more than their parents do. “They access images about life in other parts of the world on television and the Internet, and they desire to live that way,” he explains.

Geographer Robert Kates, a visiting scholar at the Harvard Center for International Development, contends that consumption rates are actually more important than population. Currently, a huge per-capita consumption disparity exists between rich and poor nations. According to the September 2003 *Population Bulletin*, published by the Population Reference Bureau, in 1999 the average North American consumed more than 15 times the energy of the average African (230 gigajoules—equivalent to about 143 barrels of oil—in North America compared with 15 gigajoules in Africa).“Most people accept the notion that major, long-term environmental problems will stem more from consumption than from population growth,” Kates says. “Population growth is one of the forces that drives consumption. But there are a whole host of other forces as well—growing income, changing diets, the creation of transnational markets.”

Kates argues that potential growth rates for consumption around the world are much greater than the better-known predicted rates for population growth. Therefore, he suggests, the number of people isn’t as important as what those people do. “The increase in the number of people is clearly slowing down everywhere in the world,” he says. “But the increase in consumption by those people is going up everywhere, except in Africa, and there’s no sign of diminution in the future. So there will be a shift from long-term historic concern about population to a growing concern about how, what, and where we consume.”

Others, however, say while paying attention to consumption is indeed a critical force, its importance should not sideline the question of where and at what level population growth will end. “If our [global] population had stabilized where it was in antiquity, at about two hundred fifty to three hundred million, our consumption probably wouldn’t make too much difference,” says Engelman. “But it’s precisely because human population has gone where it is that consumption has the global impacts that it has. How much ‘environmental space’ each of us has to consume sustainably has everything to do with how many of us there are.”

## The Impact of Population

Whether one chooses to attribute impacts to human numbers or human behavior, the fact remains that the world’s population—its numbers, its movement, its actions—is having a profound impact on human and environmental health. A variety of organizations and individuals, including the UN and other international agencies, nongovernmental organizations, scientists, and demographers, have identified many of the ways in which this is happening.

### Water availability.

Engelman points out that the amount of fresh water on Earth is roughly the same today as it was 3,000 years ago, while population has increased 40-fold. Declining water tables are a growing problem in much of the world. According to the Population Reference Bureau, 12 of the world’s 15 water-scarce countries are in the Middle East and North Africa, comprising an area that experienced more than a doubling of population—from 173 million to 386 million people—between 1970 and 2001. Growing additional food to nourish growing populations will rely heavily on irrigation, placing greater strain on water tables. The Millennium Ecosystem Assessment reports that usage levels of fresh water for drinking, industry, and irrigation are “unsustainable.” The American Association for the Advancement of Science (AAAS), in its 2000 *AAAS Atlas of Population and Environment*, predicted that the situation is “likely to be worsened by the deteriorating quality of water, polluted by industrial wastes and sewer discharges.”

### Deforestation.

According to the Population Reference Bureau, human activities during the 1990s resulted in the deforestation of 563,709 square miles of land, roughly the equivalent of Colombia and Ecuador combined. Most of the deforestation occurred in Africa and South America, where forests have been cleared for cropland, fuel use, and commercial sale of wood products. The environmental and human health impacts of deforestation are varied, including increased propensity for flooding, loss of medicinal species and fuel wood, soil erosion, and exacerbation of climate change as carbon is released back into the atmosphere. Related to deforestation is the issue of biodiversity loss. The World Conservation Union estimates that nearly one-fourth of the mammals and one-eighth of the birds on Earth are now threatened with extinction.

### Fisheries.

“The fishery story is a sad case of overuse by humans,” says Bongaarts. “Fish populations have collapsed in many parts of many oceans, and lower-quality fish are replacing them.” According to the AAAS atlas, the world’s marine catch increased fivefold between 1950 and 1990, but has remained stagnant ever since. The Millennium Ecosystem Assessment took an even bleaker view, finding that harvests have been declining since the late 1980s (Reid says the discrepancy relates to how one interprets the official statistics reported by different countries).

### Climate change.

The link between population growth and climate change is less clear. Engelman points out that the vast majority of climate change is driven by emissions from industrialized countries, the populations of which will soon peak or have already done so. But poor countries are rapidly expanding their industrial capacity in response to out-sourcing by industrialized countries, and their share of climate change–related emissions will increase rapidly in coming years, raising the need for international agreements on emissions reductions, Engelman says.

### Air quality.

The World Health Organization (WHO), in its 1999 *Air Quality Guidelines*, said that outdoor air pollution in Western Europe and North America has improved since 1970, but in less developed countries air pollution in the large cities—including Delhi, Jakarta, Mexico City, and many Chinese cities—is severe. So is its impact on public health. The World Resources Institute studied the health effects of air pollution in cities in poor nations and said in the 1999 report *Urban Air Pollution Risks to Children: A Global Environmental Health Indicator* that it was responsible for 50 million cases per year of chronic cough among children under age 14.

### Infectious disease.

Human population growth and migration has also fostered the emergence of many infectious diseases by increasing population density. This is especially true in urban areas, where illnesses such as dengue and cholera are becoming more common, the Population Reference Bureau reported in the September 2003 *Population Bulletin*. Encroachment into wildlife habitats also exposes humans to new diseases. “Increased contact with wildlife and associated diseases, combined with international trade in livestock, has led to outbreaks of diseases such as rinderpest [a viral disease affecting ungulates] in Africa and foot-and-mouth disease in Europe,” stated the report.

## The U.S. Situation

The Center for Environment and Population (CEP), a nonprofit research and public policy organization, will be releasing a national report this fall that will explore the relationship between U.S. population trends and their impact on health and the environment. Victoria Markham, director of the CEP and executive editor of the *AAAS Atlas of Population and Environment*, says one of the reasons for the study is that the United States, in a departure from other industrialized nations, is experiencing significant population growth and will continue to do so.

Where the AAAS atlas was one of the first large efforts to tie known data about environmental change to population, the upcoming CEP report will do the same sorts of comparisons within American borders. Markham says the latter report will focus on several human population variables that relate to environmental impact—population growth, distribution, movement, and makeup, as well as household demographic trends and consumption rates—and apply them to the nation’s four census regions.

With a population of 298 million, the United States is the third most populous country in the world, behind China (population 1.3 billion) and India (population 1.1 billion). Projections in the Population Reference Bureau’s *2004 World Population Data Sheet* call for the United States to remain third behind China and India for decades to come, while two other current industrialized countries, Russia and Japan, will be dropping out of the top 10 and leaving the United States as the only currently industrialized country on that list by 2050.

“Couple our growing population with our disproportionately high rate of resource consumption, and you have a volatile combination,” Markham says. “The United States turns out to be a world leader in terms of per-capita global environmental impact.”

Like the rest of the world, the United States is becoming ever more urbanized, but at a more advanced level, as 80% of Americans now live in metropolitan areas, according to the 2001 U.S. Census Bureau report *Population Change and Distribution, 1990 to 2000*. But while more Americans than ever are living in metro areas, most of the growth is occurring outside center cities, in outlying suburban areas.

Markham says this outcome—sprawl—can be illustrated by the fact that while the American population has grown by 17% in the last two decades, the land area converted to metropolitan use grew by 50%. “Air pollution is very closely tied to population,” she says. “Transportation is the fastest growing energy-use sector in the United States, and it’s particularly tied to this sprawled development because people have to drive more and drive farther. The result is increased carbon dioxide emissions.”

Another trend is a continuing higher rate of population increase in the South and West, compared with the Midwest and Northeast. This trend largely reflects the movement of the industrial infrastructure from the North to the South and West starting in the 1960s for various economic reasons, such as lower taxes and lower labor costs. In terms of environmental impact, the population growth in the West is especially worthy of concern because of the region’s fragile water supply. “Population growth couldn’t be happening in a more environmentally vulnerable place in the United States,” Markham says. The Ogallala aquifer, which lies under eight western states and is the largest groundwater system in North America, accounting for 20% of all irrigated land in the United States, is down one-third of its capacity and is shrinking at the rate of a foot per year, according to Markham.

Meanwhile, Americans are living in ever larger per-capita household space, which exacerbates energy consumption. The CEP report will describe the continuing decline in number of persons per household, which translates into more households. At the same time, the physical size of American homes is growing ever larger. According to Markham, the proportion of houses of at least 3,000 square feet more than doubled between 1988 and 2003; during that same time, the number of new houses smaller than 1,200 square feet declined. And lot sizes of new one-family houses outside the country’s metropolitan areas rose by 6% in the past 10 years, according to the Census Bureau. The increase in the number of houses overall combined with larger lot sizes means more land is being used for residential development than ever before.

## Reasons for Hope, Possible Futures

Discussions about burgeoning human populations and their impact on health and the environment abound in gloomy data and prospects of doom. But experts also suggest there are reasons to be somewhat optimistic. First, they say, humanity has proven itself to be more resourceful than Malthus and Ehrlich gave it credit for being. “Basically, forty or fifty years ago, the whole world was growing rapidly,” Bongaarts says. “There was a huge concern about potential food shortages and environmental problems. But birth rates have declined, so growth is not as rapid as people thought it would be.”

Even though the rates are declining in poor countries, they’re still higher than the acknowledged replacement figure of 2.1 children per woman. Still, Asian, Latin American, and Caribbean women are bearing children at a rate of 2.6 children per woman in 2004 compared to about 5 per woman in 1970, according to the UN Population Division. African women still have 5 children on average, but that’s down from 6.7 in 1970. Europe has dropped from 2.2 children per woman to a population-slashing 1.4. In the major world regions, only North America has not seen declining birth rates. North American women averaged 2.0 children in 1970 and the figure was the same in 2004.

To many observers, the decline in global birth rates is clear proof of the effectiveness of family planning programs. “I think the greatest proportion of demographic research points to the worldwide effort to make contraception available, which was clearly desired and was in fact picked up and used,” Engelman says.

Lars Bromley, a senior program associate in the AAAS Office of International Initiatives, has come to the same conclusion. “If a country works to reduce its birth rate, it’s not a foregone conclusion that they’re destined to have twelve children per woman,” he says. “Places like Bangladesh and elsewhere have really performed miracles over the last generation.” According to *Bangladesh Demographic and Health Survey 2004*, published by the U.S. Agency for International Development, birth rates in that country have declined from 6.3 children per woman in the early 1970s to 3.0 children in 2004.

Another improvement, Kates points out, is that although the total amount of energy consumed continues to rise, the world is reducing its “energy intensity”—that is, the amount of energy it uses per unit of production—at a rate of about 1% per year. This is mostly due to improved energy-saving technology.

But as the scientists who conducted the Millennium Ecosystem Assessment conclude, a broad international response is necessary to deal with the environmental declines caused by increasing human pressure. They didn’t make predictions about what may happen, but they did offer four possible future scenarios. The first, “Global Orchestration,” depicts a world that makes economic development a priority and emphasizes solving environmental problems rather than preventing them in the first place. The second, “Order from Strength,” represents a fragmented world concerned primarily with security and protection, where the approach to the environment again is reactive. The third, “Adapting Mosaic,” would deemphasize economic development and put priority on the health of ecosystems, largely through the strengthening of local management strategies. The fourth scenario, “TechnoGarden,” describes a future in which a unified world relies on environmentally sound technology and highly managed, often engineered, ecosystems to deliver ecosystem services, and that achieves both strong economic growth and a healthier world.

Reid believes that the work on which direction the world should go must start soon. And he believes the debate must focus more on population than it has to date. “Population is one of those issues that’s so central and so politicized,” he says. “Sometimes you worry that people are ignoring it because of the political side of it, but it’s critical that people keep thinking about it and about steps that can be taken to address population problems.”

## Signs of Ecological Change

Over the past century and especially over the past 40 years, people have effected vast changes in the global environment. Those people most directly affected by environmental challenges, from water pollution to climate change, are also the poorest and least able to change livelihoods or lifestyles to cope with or combat ecological decline. Some signs of ecological change include:

**Source:** UNFPA. 2004. State of World Population 2004: The Cairo Consensus at Ten—Population, Reproductive Health and the Global Effort to End Poverty. New York, NY: United Nations Population Fund.

## Figures and Tables

**Figure:**
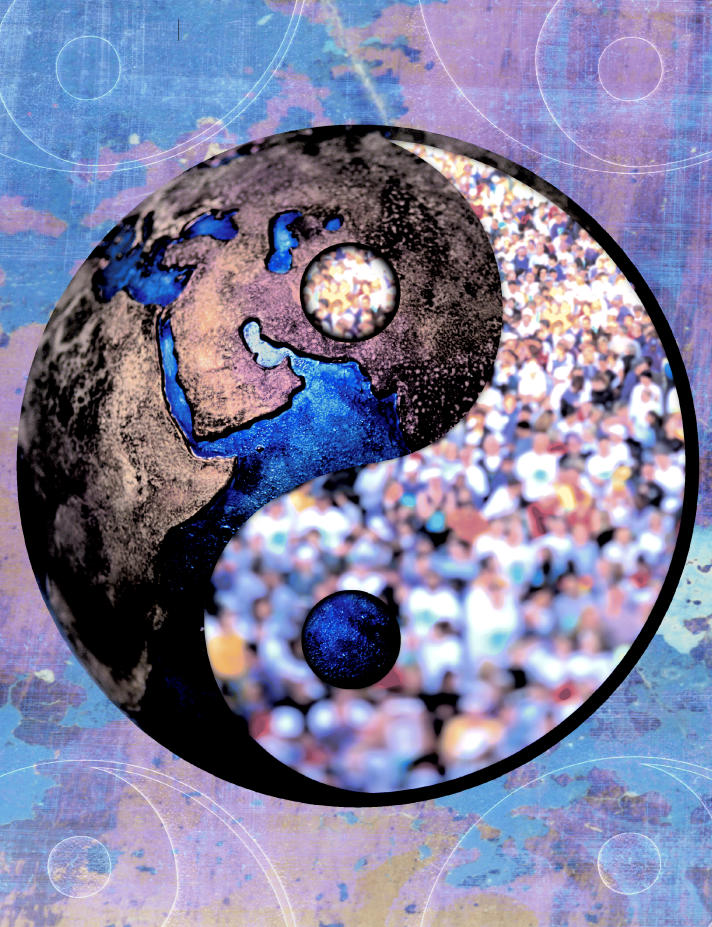


**Figure:**
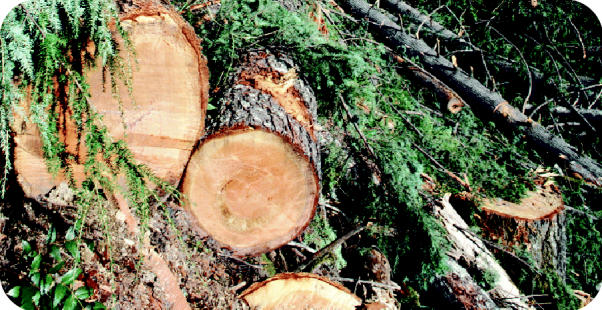
Deforestation. Farmers, ranchers, loggers, and developers have cleared about half the world’s original forest cover, and another 30% is degraded or fragmented.

**Figure:**
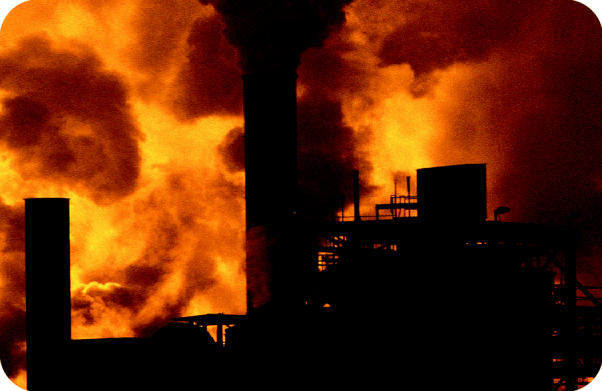
Climate change. As a result of fossil fuel consumption, carbon dioxide levels today are 18% higher than in 1960 and an estimated 31% higher than at the onset of the Industrial Revolution in 1750. Accumulation of greenhouse gases (including carbon dioxide) in the atmosphere is tied to rising and extreme change in temperatures as well as more severe storms.

**Figure:**
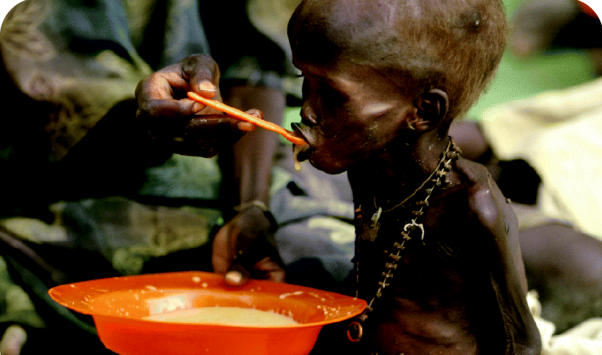
Food insecurity. Over the past half-century, land degradation has reduced cropland by an estimated 13% and pasture by 4%. In many countries, population growth has raced ahead of food production in recent years. Some 800 million people are chronically malnourished, and 2 billion lack food security.

**Figure:**
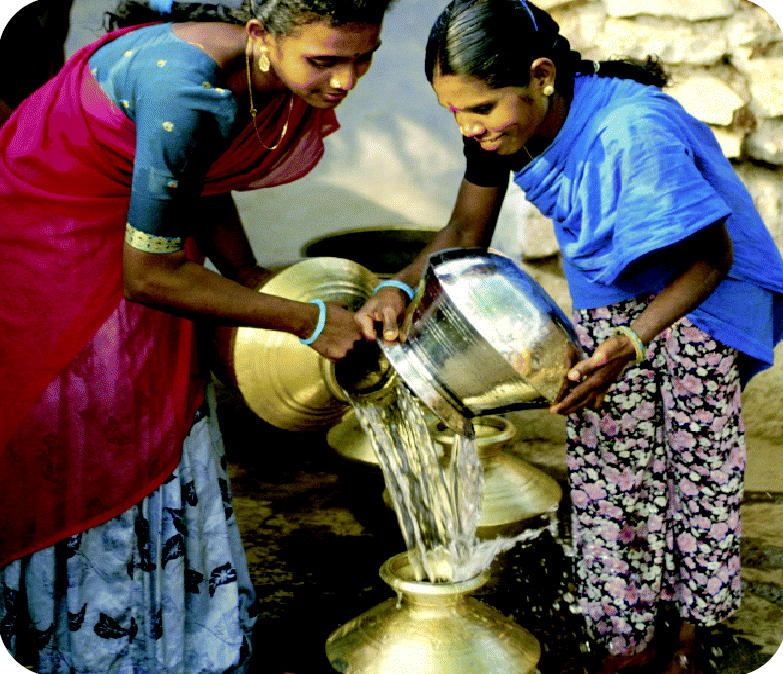
Water scarcity. Since the 1950s, global demand for water has tripled. Groundwater quantity and quality are declining due to overpumping, runoff from fertilizers and pesticides, and leaking of industrial waste. Half a billion people live in countries defined as water-stressed or water-scarce; by 2025, that figure is expected to surge to between 2.4 billion and 3.4 billion.

**Figure:**
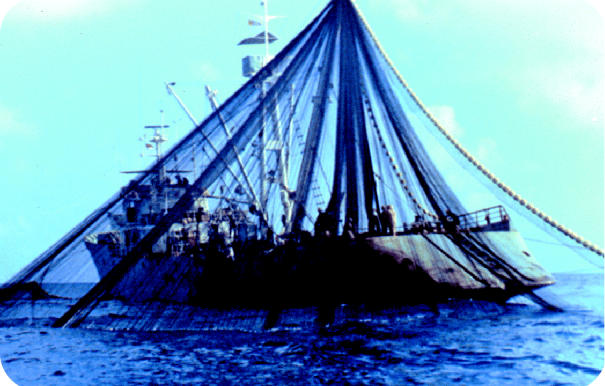
Overfishing. Three-quarters of fish stocks are now fished at or beyond sustainable limits. Industrial fleets have fished out at least 90% of large ocean predators—including tuna, marlin, and swordfish—in the last 50 years.

**Figure:**
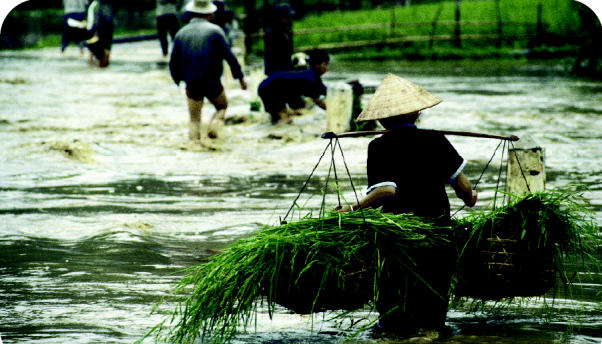
Sea level rise. Sea level has risen an estimated 10–20 centimeters, largely as a result of melting ice masses and the expansion of oceans linked to regional and global warming. Small island nations and low-lying cities and farming areas face severe flooding or inundation.

**Figure:**
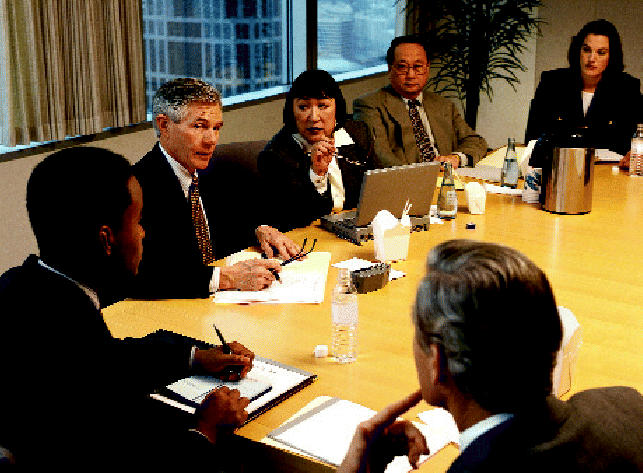
Globally based trade reform lifts populations out of poverty, freeing up resources to respond to environmental problems as they become apparent.

**Figure:**
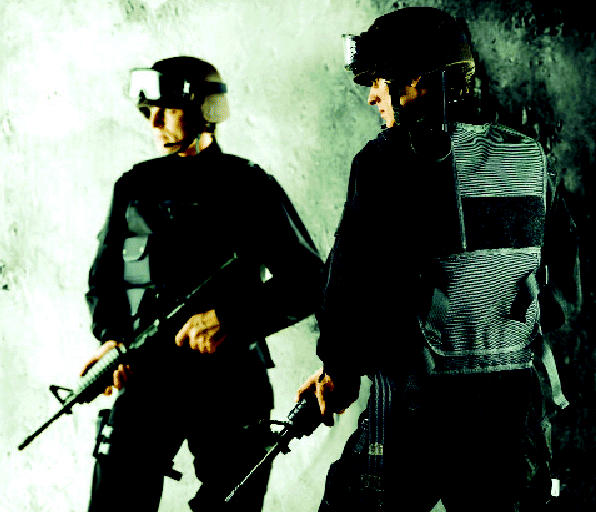
Nations are concerned primarily with security. Powerful countries shift burdens to weaker nations, and ecosystem services become increasingly vulnerable.

**Figure:**
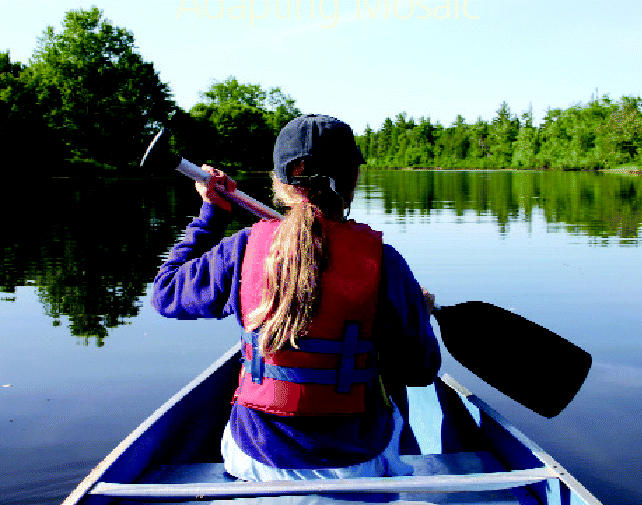
Political activity targets regional ecosystems, and investments are geared toward better understanding of these systems. Some areas thrive while others continue to degrade.

**Figure:**
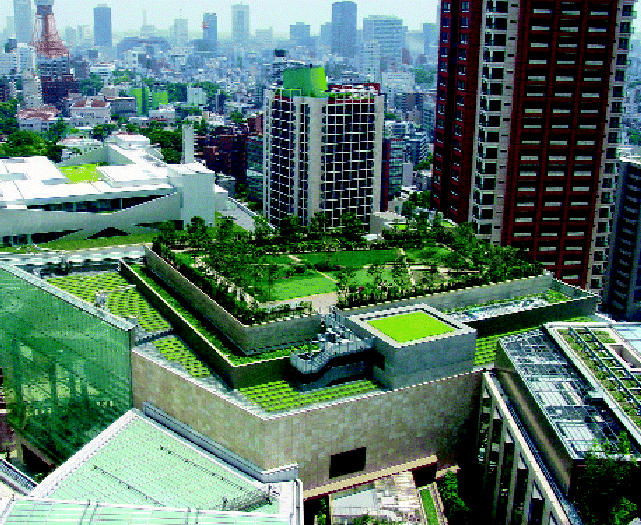
A globally connected world relies on highly managed ecosystems to provide services and solutions to environmental problems. Ecological engineering flourishes.

**Figure:**
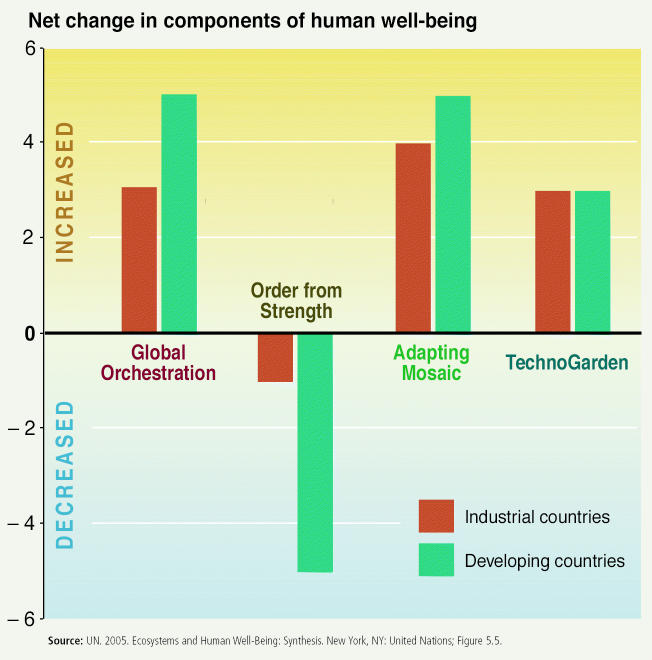
The Millennium Ecosystem Assessment examined how each scenario would increase or decrease material well-being, health, security, social relations, and freedom of choice and action.

**Table:** Countries Accounting for About 75% of the World Population by Order of Population Size

**1950**			**2005**			**2050**		
**Rank**	**Population^a^**	**Cumulated %**	**Rank**	**Population^a^**	**Cumulated %**	**Rank**	**Population^a^**	**Cumulated %**
1. China	555	22.0	1. China	1,316	20.4	1. India	1,593	17.5
2. India	358	36.2	2. India	1,103	37.4	2. China	1,392	32.9
3. United States	158	42.5	3. United States	298	42.0	3. United States	395	37.2
4. Russia	103	46.6	4. Indonesia	223	45.5	4. Pakistan	305	40.6
5. Japan	84	49.9	5. Brazil	186	48.4	5. Indonesia	285	43.7
6. Indonesia	80	53.0	6. Pakistan	158	50.8	6. Nigeria	258	46.6
7. Germany	68	55.7	7. Russia	143	53.0	7. Brazil	253	49.4
8. Brazil	54	57.9	8. Bangladesh	142	55.2	8. Bangladesh	243	52.0
9. United Kingdom	50	59.9	9. Nigeria	132	57.3	9. Dem Rep Congo	177	54.0
10. Italy	47	61.7	10. Japan	128	59.2	10. Ethiopia	170	55.9
11. France	42	63.4	11. Mexico	107	60.9	11. Mexico	139	57.4
12. Bangladesh	42	65.0	12. Vietnam	84	62.2	12. Philippines	127	58.8
13. Ukraine	37	66.5	13. Philippines	83	63.5	13. Uganda	127	60.2
14. Pakistan	37	68.0	14. Germany	83	64.8	14. Egypt	126	61.6
15. Nigeria	33	69.3	15. Ethiopia	77	66.0	15. Vietnam	117	62.9
16. Spain	28	70.4	16. Egypt	74	67.1	16. Japan	112	64.1
17. Mexico	28	71.5	17. Turkey	73	68.2	17. Russia	112	65.3
18. Vietnam	27	72.6	18. Iran	70	69.3	18. Iran	102	66.5
19. Poland	25	73.6	19. Thailand	64	70.3	19. Turkey	101	67.6
20. Egypt	22	74.4	20. France	60	71.2	20. Afghanistan	97	68.7
			21. United Kingdom	60	72.2	21. Kenya	83	69.6
			22. Italy	58	73.1	22. Germany	79	70.4
			23. Dem Rep Congo	58	73.9	23. Thailand	75	71.3
			24. Myanmar	51	74.7	24. United Kingdom	67	72.0
						25. Tanzania	67	72.7
						26. Sudan	67	73.5
						27. Colombia	66	74.2
						28. Iraq	64	74.9

a In millions.

**Source:** UN. 2005. World Population Prospects: The 2004 Revision. Highlights. New York, NY: United Nations; Table VIII.3.

**Table:** Countries Accounting for About 75% Average Annual Population Increase in the World

**1950–1955**			**2005–2005**			**2045–2050**		
**Rank**	**Pop Increase^a^**	**Cumulated %**	**Rank**	**Pop Increase^a^**	**Cumulated**	**Rank**	**Pop Increase^a^**	**Cumulated %**
1. China	10,849	22.8	1. India	16,457	21.7	1. India	4,994	14.8
2. India	7,507	38.6	2. China	8,373	32.7	2. Dem Rep Congo	2,935	23.5
3. United States	2,652	44.1	3. Pakistan	3,057	36.8	3. Uganda	2,855	32.0
4. Brazil	1,782	47.9	4. United States	2,812	40.5	4. Nigeria	2,523	39.5
5. Russia	1,740	51.5	5. Nigeria	2,784	44.2	5. Pakistan	2,498	46.9
6. Indonesia	1,382	54.5	6. Indonesia	2,721	47.7	6. Ethiopia	1,999	52.8
7. Japan	1,238	57.1	7. Bangladesh	2,581	51.1	7. Afghanistan	1,699	57.9
8. Bangladesh	852	58.8	8. Brazil	2,509	54.5	8. Bangladesh	1,493	62.3
9. Pakistan	837	60.6	9. Ethiopia	1,781	56.8	9. United States	1,489	66.7
10. Mexico	800	62.3	10. Dem Rep Congo 1,499	58.8		10. Kenya	1,058	69.9
11. Nigeria	750	63.9	11. Philippines	1,458	60.7	11. Niger	1,007	72.9
12. Philippines	645	65.2	12. Mexico	1,388	62.5	12. Yemen	881	75.5
13. Thailand	627	66.5	13. Egypt	1,349	64.3			
14. Turkey	625	67.8	14. Afghanistan	1,226	65.9			
15. Egypt	572	69.0	15. Vietnam	1,113	67.4			
16. Ukraine	560	70.2	16. Turkey	992	68.7			
17. Vietnam	537	71.4	17. Uganda	901	69.9			
18. South Korea	513	72.4	18. Iraq	747	70.9			
19. Poland	491	73.5	19. Kenya	713	71.8			
20. Iran	435	74.4	20. Tanzania	713	72.8			
			21. Colombia	696	73.7			
			22. Sudan	666	74.6			
**WORLD**	**47,586**	**100.0**	**WORLD**	**75,835**	**100.0**	**WORLD**	**33,697**	**100.0**

a In thousands.

**Source:** UN. 2005. World Population Prospects: The 2004 Revision. Highlights. New York, NY: United Nations; Table VIII.6.

